# A Review on Microstructural Features and Mechanical Properties of Wheels/Rails Cladded by Laser Cladding

**DOI:** 10.3390/mi12020152

**Published:** 2021-02-04

**Authors:** Xinlin Wang, Lei Lei, Han Yu

**Affiliations:** School of Mechanical Engineering, Dalian Jiaotong University, Dalian 116028, China; wxl_me@djtu.edu.cn (X.W.); 18340879580@163.com (H.Y.)

**Keywords:** rail, laser cladding, microstructural characteristics, mechanical properties

## Abstract

The service life of rails would be remarkably reduced owing to the increase of axle load, which can induce the occurrence of damages such as cracks, collapse, fat edges, etc. Laser cladding, which can enhance the mechanical properties of the rail by creating a coating, has received great attention in the area of the rails due to the attractive advantages such as low input heat, small heat-affected zone, and small deformation. In this paper, recent developments in the microstructural characteristics and mechanical properties of a cladded layer on the rail are reviewed. The method of process optimization for enhancing the properties of a cladded layer are discussed. Finally, the trend of future development is forecasted.

## 1. Introduction

The rail, which is a major component to support the running of trains, has a significant effect on the stationarity and safety during the train working. With the rapid development of high-speed and heavy haul trains which means the rail will carry heavier frictional loads, the surface damages of rail, i.e., wear and rolling contact fatigue (RCF), etc., have become seriously severe. The surface damages often occur and accumulate at the contact surface between the rail–wheel components during the service lives, which also are the main reasons for preventative maintenance in the modern railway infrastructure [1,2,3]. Therefore, in order to promote the development of transportation industry and the safety of the train running, how to prolong the service life of the rail and repair the damaged rail have become an urgent task.

Wear and RCF are the two main factors that have a significant effect on the durability of the rail [4,5]. In order to obtain superior wear and RCF resistance, heat treatment is usually used in the manufacturing process of the rails. However, this additional step would lead to an increased cost [6]. The traditional techniques, such as case hardening and peening, are selectable solutions to enhance the wear and RCF performance of many engineering materials. However, the length of rail is usually beyond 100 m, which means it is impractical and costly to adopt those traditional techniques into the manufacturing of rails. Therefore, forming a coating with excellent wear and RCF resistance on the surface of rails becomes an appropriate method. However, owing to the occurrence of the maximum shear stress, which is a dominating driver of RCF below the surface of the material, the coatings created by traditional surface coating technology, such as thermal spraying, are not sufficiently thick to prevent damage caused by the sub-surface shear stress at its lowest depth [7]. On the other hand, for the damaged rail, two main ways, which include replacing directly and repairing the damaged rail, have been widely used [8]. Although the process of replacing is simple and easy, replacing the damaged rail would not only aggravate the waste of resource and energy but also increase risk for the running safety of the train because of the increased number of splices between the rails [9,10,11]. The repairing of the damaged rail means repairing online using kinds of technology without excising the damaged rail. Repairing can abolish the process of the excision of damaged rail and welding of splice that can reduce the waste of resource and energy. In addition, the “window” time can be averted that assures the normal running of the train [12]. A proper repair contributes to the improvement of the surface quality of the rail, the prolonging of the service life, as well as the increase of its economic value and social benefits. At present, the technology for repairing the damaged rail contains wheel–rail lubrication, rail grinding, rail mailing, surface coating, etc. [13]. The surface coating technology, such as thermal spraying, plasma arc surfacing, and laser cladding not only can repair the local damage but also improve the mechanical properties and prolong the service life. Compared to the conventional technology, the laser cladding becomes more and more noticeable for the rail repairing, owing to the advantages such as low input heat, small heat-affected zone, small deformation, and the ability to create the metallurgical bonding between the clad and substrate [14].

**Figure 1:**
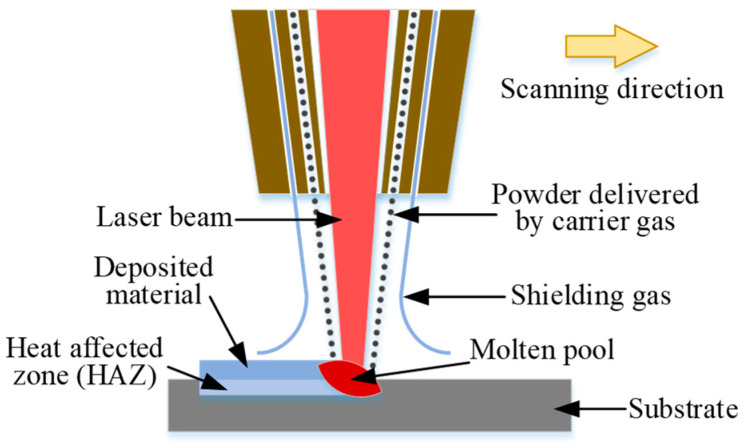
Schematic diagram of laser cladding process.

Figure 1 shows the schematics illustration of the laser cladding process. At the beginning, the substrate is melted by a high-powered laser beam to create a molten pool. The delivered metallic powder carried by a flowing inner gas (such as Argon) is injected into the molten pool simultaneously. The powder captured by the molten pool melts which contributes to the increased volume of the molten pool. As the powder nozzle and laser beam move above the substrate under the guidance of computer, the clad layer is deposited on the substrate [15,16].

In terms of the appropriate cladding material for wheel–rail contact, the excellent wear resistance and great suitability for laser and load bearing capability are the main properties to consider. A number of hardfacing materials, which are capable of providing excellent wear resistance and preserving the surface integrity of surfaces, have been selected as the potential materials mainly including austenitic stainless steel (i.e., 316 L, 410, and 420), martensitic stainless steel, Co-based alloy (i.e., Stellite 6, Stellite 12, and Stellite 21), Ni-based alloy, Ti, and TiB_2_. Table 1 provides a summary of some common materials that have been investigated for the repairing and cladding of rail by laser cladding. With the changes of the cladded materials, processing parameters, and the treatment during the laser cladding, the cladded layers would present different characteristics. The aim of this article is to provide an overview of the microstructural features and mechanical properties of the clad created on the rail.

## 2. Effects of Process Parameters on Geometrical and Microstructural Characteristics

There are many laser cladding process parameters that have a significant effect on the properties of the cladded layer by affecting the geometric features, microstructures, and residual stress, which are dominated by the thermal history during the laser cladding process, such as laser power, powder feed rate, scanning speed, number of layers, as well as the terms of powder materials, etc. [33]. With the changing of process parameters, the input energy, shape of molten pool, cooling and solidification rate, local thermal gradients, and heat transfer will be completely changed [34,35]. Through affecting the thermal history during the laser cladding process, those parameters will have significant impacts on the geometry, microstructures, and mechanical characteristic of the clad.

### 2.1. Geometrical Features of the Clad

Based on the investigation by the previous researchers, the following conclusions about the effects of process parameters on the clad characteristics can be drawn: (1) The width of the clad increases with the increase of the laser power and powder feed rate whereas it decreases with the increase of scanning speed; (2) The height of clad is mainly affected by the scanning speed and powder feed rate; (3) When the powder feed rate is low, the increase of the scanning speed will increase the height of the clad; (4) The increase of the laser power contributes to the increase of the geometric size of the clad as well as the creation of the fine grain. However, the increase of the scanning speed would lead to the decrease of the geometric size of the clad and the appearance of the coarse grain; (5) The powder feed rate has a significant effect on the height and depth of the clad [36,37,38]. In the research work from Lai et al., a defect-free surface of a 410 L cladded layer was produced by using the laser power of 3200 W, powder feed rate of 3 RPM, and scanning speed of 1000 mm/min, regardless of the number of cladded layers. However, the evaporation of the cladding materials will occur if the scanning speed comes up to 1200 mm/min. For the Stellite 6 cladding, the heating problems associated with heating bubbles were alleviated by increasing the scanning speed from 1000 to 1200 mm/min. When the SS420 and Stellite 21 cladding were processed at 1000 mm/min and 1200 mm/min, the surfaces of the cladded layers were free of heating bubbles, which implied that heating problems did not occur [3]. The 410 L deposited layer with the transverse cladding direction contained a large portion of the fine dendritic morphology, whereas the ferrite was presented on the surface of the deposited layer with a longitude cladding direction that was attributed to the combined effect of cooling rate and dilution of the substrate because of the cladding direction [3].

### 2.2. Microstructure

The microstructural features of clad, such as grain size and morphology, are dominated by the thermal history, which is strongly sensitive to the processing parameters during the laser cladding. The solidified microstructure is decided by the temperature gradient at the solid–liquid interface (G), solidification rate of the molten pool, and the ratio of cooling rate to thermal gradient (R). The ratio of G to R (G/R) can affect the shape of the solid–liquid interface, and the cooling rate, which is represented as G × R, would control the dimensions of the microstructure [34,39,40]. The early stage of laser cladding is accompanied by the large value of G/R that contributes to the appearance of the columnar crystal. With the laser cladding continuing, the value of G/R would decline, which prompts the formation of dendrite crystal [22,41,42,43].

When the clad was deposited on the rail by laser cladding with a high scanning speed (400–800 mm/min), the fine grain can be generated because of the significant temperature gradients, which contributes to the high heating and cooling rate of the molten pool [29]. However, the brittle martensitic structures with hardness of HV800–1000, which is harmful to the mechanical properties, would be formed in the heat-affected zone (HAZ), and the organization is more complicated in the transition bond [26,30]. The overview images of all microstructures would show a HAZ dilution zone, coarse-grained zone, and fine-grained microstructure zone, followed by an inter-critical or sub-critical HAZ adjacent to the base material based on the difference of the substrate [7]. The region near the boundary between the cladded layer and substrate presented columnar dendrites with different orientations, which were attributed to heat accumulation caused by remelting between layers. However, the cellular dendrites were found in the middle of coatings and around the top surface, in which the grain size is smaller [18,30]. In addition, the grain size in the HAZ was much smaller than those in the base materials. This phenomenon might be explained by the fact that the top region of the base materials undergoes a quenching process owing to the drastic heating-cooling cycles during the laser-cladding process [18].

For the Ni-based alloy clad, the cladded layers showed the presence of both borides and mixed carbides. The processing conditions, which include a low powder feed rate, lead to the increase of the specific energy and the reduction of the cooling rate, contributing to the growth of leaflike microstructures. Excessively, a higher laser power and powder feed rate would restrict the growth of leaflike microstructures and promote the appearance of a more angular microstructure, which would provide higher hardness [29]. The investigation reported by Li et al., in which the Ni-based alloy is deposited on a on U71Mn rail using a 6 kW continuous wave fiber laser (YLR-6000, IPG), revealed that the HAZ of specimen by laser cladding is composed of small acicular martensite. However, the HAZ of specimen by laser-induction hybrid cladding is composed of pearlite, whose interlamellar spacing is much smaller than that of the rail substrate. The different microstructure transformations of the HAZs contributed to the different thermal cycles of the molten pools and HAZs in different processes. They also reported that the coatings are mainly composed of γ phase with a face-centered cubic (FCC) crystal structure and Cr_23_C_6_ with a cubic crystal structure [26].

For the Co-based alloy clad, the microstructures, including Co-rich primary dendrites, eutectic, and interdendritic regions with hard carbides associated with W, Cr, and Co elements, would be obtained. Huo-ming Guo investigated the microstructure and wear behavior of a laser cladding Co-based alloy coating on single wheel or rail material. In addition, the coating consists of dendrite and eutectic [30]. Wang deposited the Co-based alloy powder on the wheel/rail rollers using a multimode cross flow CO_2_ laser (TR-3000) and found that the cladding coatings are composed of γ-Co phase and carbide Cr_23_C_6_ created by the Cr element, which is affluent in Co-based alloy powders, and the C element forms the carbide Cr_23_C_6_ by means of chemical reaction at high temperature during the laser-cladding process [31]. Lai et al. cladded the Stellite 6 and Stellite 21 on the rail substrate and obtained the clad in which the microstructures presented Co-rich primary dendrites and interdendritic regions with hard carbides associated with W, Cr, and Co elements [44]. The Co-rich dendrite, eutectics of Cr, Co, and W carbides, as well as the Co-based matrix were observed in the Stellite 6-cladded layer and the tungsten-rich phase presented at the bright regions in the interdendrites [45,46].

The microstructures of Fe-based alloy and titanium alloy clad were investigated as well. Lai et al. deposited the 410 L and 420SS on the rail substrate. For the 410 L deposition, the microstructural characteristics show the colonies of ferrite inside a martensitic matrix [3,40]. The single 420SS deposits presented a significant portion of fine martensitic dendrites. However, three dendritic morphologies including equiaxed, columnar/cellular, and planar dendrites were shown in the double 420SS deposits through the cladding’s thickness, which was affected by the temperature gradient and the growth rate of solid–liquid interface [3,42,47]. Lu et al. deposited a grade of martensitic stainless steel (MSS) on the European standard grade rail steel R260 and a lower grade rail steel R200 [7]. Z.K. Fu et al. investigated the influence of laser cladding Fe-based alloy on microstructure of wheel/rail materials. The results indicated that the coating, which is composed of (Fe, Ni) solid solution and Cr_7_C_3_, consists of dendrites and eutectic [22]. Aladesanmi et al. pointed out that the bright alpha phase would be notable with more titanium presence by conducting the laser cladding experiments in which the mix ratio of Ti and TiB_2_ is different [32].

### 2.3. Microhardness

**Figure 2:**
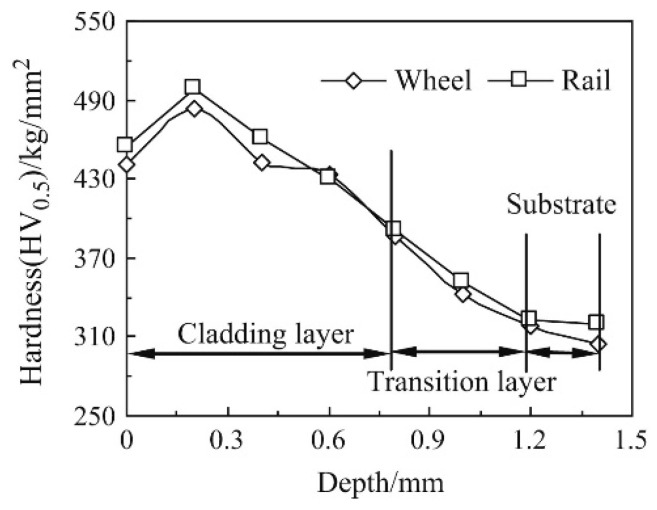
The change of hardness along with the depth direction of the wheel/rail rollers cladded by Co-based alloy powders (reprinted from ref. [31], copyright (2014), with permission from Elsevier).

The surface hardness of a wheel and rail without laser cladding is lower than that of a wheel/rail treated by laser cladding. In addition, the surface hardness of the rail is larger than that of the wheel, regardless of whether they undergo the laser cladding or not [31]. With an increase of depth, the hardness of the cladding coatings created using a multimode cross flow CO_2_ laser (TR-3000) decreases gradually along the cladding layer to the transition layer and is close to the substrate hardness of the wheel/rail, as shown in Figure 2 [31].

**Figure 3:**
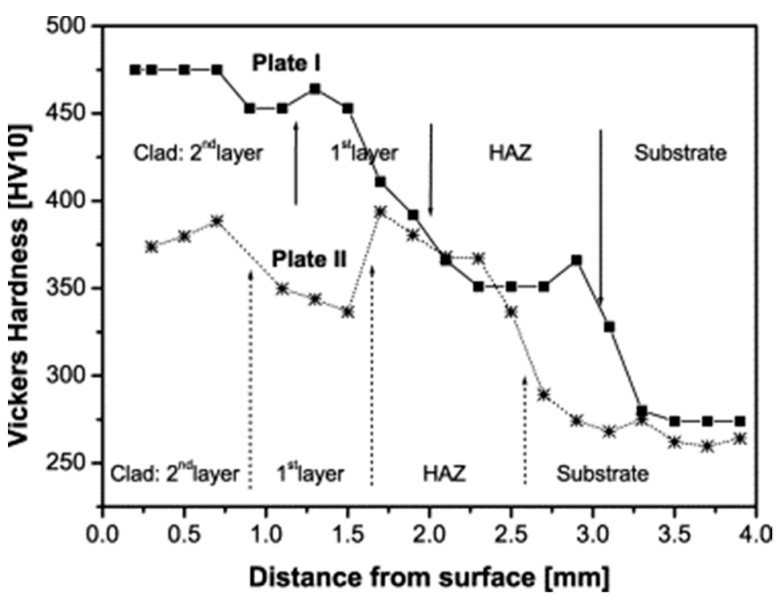
Hardness evolution as a function of the depth below the clad surface for both carbon steel (B 82) plates cladded Co-Cr alloys (Reprinted from ref. [48], copyright (2004), with permission from Elsevier).

Niederhauser et al. cladded two successive layers with Co-Cr alloys on the carbon steel (B 82). Through controlling the laser energy input and scanning speed to change the thermal history during the laser cladding process, two plates, which were named Plate I and Plate II with clad thicknesses of 2 mm and 1.6 mm, respectively, were obtained. As shown in Figure 3, the hardness values differ strongly from Plate I to Plate II. However, the values of hardness were very similar in both the unaltered substrate material as in the heat-affected zone for both plates. The results indicated that laser cladding was not very susceptible to small changes in the process parameters [48].

**Figure 4:**
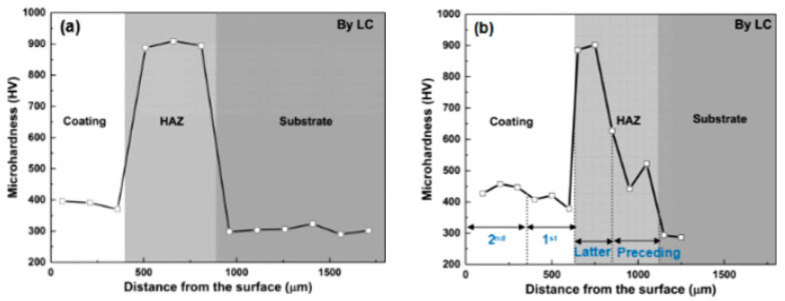
Microhardness distributions along the depth direction of (**a**) the single-layer and (**b**) two-layer Ni-based cladding track specimens prepared by LC on U71Mn rail using a 6 kW continuous wave fiber laser (YLR-6000, IPG) (Reprinted from ref. [26], copyright (2019), with permission from Elsevier).

Despite the layer number, the HAZ region of the Ni-based specimens would present much higher hardness than that of the substrate and coatings because of the existence of the martensite structure. In the two-layer multi-track cladding specimens, the hardness of the second layer is slightly higher than that of the first layer. The microhardness of the transition region was obviously higher than that of the other regions of the second layer owing to the richness of the M_x_C_y_ phases. In addition, in the HAZ region, the microhardness of the preceding layer decreased, which contributes to the tempering effect caused by the latter layer. The results indicated that the overlapping of the tracks and layers have a significant effect on the microhardness of the HAZ, as shown in Figure 4 [26]. The same results were also observed in Stellite 6 clad on the R260 rail steel reported by Clare [41]. The hardness of the HAZ region, which consists of a rich martensitic structure, was about three times the hardness of the rail steel material in which there is a concentration of the pearlitic structure [26].

Generally, the hardness of the cladded rail is expected to be about 90–95% of the parental rail. If the cladding layers are significantly harder or softer than the parental rail, the cumulative wear of the cladding layers would be lower or higher than that of the parental rail. It could lead to an uneven worn surface of the cladded layers, which could alter the dynamic interaction between rails and wheels, increase the impact loading, and bring about vibration and nose [3]. The hardness and wear behavior in the wheel-rail contact present an inverse relationship, i.e., the higher the hardness, the less the wear, as verified by Jin et al. and Lewis et al. [49,50]. Based on the investigation in which a 4 kW IPG fiber laser was used, Lai pointed that the 410 L deposits might be more suitable for cladding because of the lower hardness compared to the 420SS deposit. In case of the Co-based cladding, the average hardness of Stellite 6 and Stellite 21 cladded layers fell within the requirement of hardness that contributed to the uniform wear rate across the profile of the laser-cladded rails. Therefore, the wheel–rail contact conditions after laser cladding verged on those that occurred in the undamaged rail and wheel surfaces. However, the Stellite 21 cladded layer presented cracking at the start of the second layer [3].

### 2.4. Residual Stress

During a laser-cladding process, it is well known that the domain drawback for the mechanical properties is the residual stress, which is created accompanied by the repeated rapid heating and rapid cooling along with the moving of a high-power laser beam [51,52]. Residual stress, which could lead to worse properties in corrosion, fracture resistance, and fatigue performance of the part treated by laser cladding would be caused by thermal shrinkage because of the high cooling rate, martensitic formation in the cladding layer and HAZ, and different coefficient of thermal expansion between the substrate and cladding material [19,48]. The tensile residual stress has a detrimental effect on the performance of the component because it reduces the effect fatigue and tensile properties of the structure. However, the compressive stress could increase the fatigue life of a component [53,54]. The results of a fatigue test from S. Niederhauser showed that the mean stress at half lifetime approached zero at the strain values of about 0.6%, which means the stresses stem from residual stresses [48].

In the interaction of wheel and rail, rail fatigue failure occurs when the combination of internal residual stresses and rail–wheel contact stress becomes critical [19]. Narayanan et al. cladded martensitic steel on the pearlitic steel (UIC 900 A/grade 260) and investigated the residual stress using a semi-destructive center hole, deep hole drilling, and non-destructive neutron diffraction techniques and pointed out that the region in the clad and near the interface presents a triaxial compressive residual stress, whereas tensile stress is showed in the substrate [25]. Roy et al. investigated the residual stress of the 401 L and Stellite 6 coating on the rail grade R400HT by laser cladding using a 4 kW IPG fiber laser with a coaxial head. The results indicated that the laser-cladding process contributed to the occurrence of compressive residual stress during to the rapid contraction during solidification. With the difference of the cladding material, the distribution of residual stress will be different. In the case of a 410 L cladded rail with a single layer, compressive residual stress was dominant in the cladding layer, which can be explained by the martensitic transformation within the microstructure of the cladding layer, and it was compensated by the tensile residual stresses in the HAZ. However, tensile residual stress was found dominant in the Stellite 6 cladded rail with a single layer. That tensile stress was driven by the structure of the cladding layer, which was mainly Co-Cr-Fe phase-γ with enriched cobalt in the form of carbides. Undergoing the double-layer cladding, the distribution of residual stress did not significantly change for the Stellite 6 cladded rail. However, the peak tensile residual stresses shifted to a deeper location for the 410 L cladded rail [19]. In addition, the post-cladding heat treatment was very effective in reducing the magnitude of residual stress in the cladding layer of the HAZ and the rail [19].

## 3. Mechanical Characteristics

### 3.1. Tensile and Bending Properties

Many research studies on laser cladding of rails mainly focused on the microstructural analysis, the evaluation of hardness, and the assessment of wear and RCF behaviour. However, the data are insufficient to comprehensively understand the correlation of the mechnical properties without knowing of the basic properties, such as the yeild strength and Young’s modulus of the cladded rails. Through the investigation of tensile and bending properties, the basic properties can be assessed, and that contriutes to studying the wear and rolling contact performance of laser-cladded rails [20].

Roy evaluated the tensile properties of the cladding layer, heat-affected zone, and substrate of laser-cladded hypereutectoid steel rails (HE400) using 410 L, SS420, and Stellite 6 powders. Based on the experimental results as shown in Table 2, the cladding layers would present similar or higher yield strength and UTS but lower elongation values than the non-cladded rail steel, regardless of the cladding material. Overall, in the longitudinal and transverse directions, the specimens in the cladding layer and HAZ presented comparable tensile properties, which demonstrated that they are isotropic in terms of plastic and elastic deformation. However, the elongation showed significant different values between the longitudinal and transverse directions, regardless of the cladding layer or HAZ specimens, which indicates certain anisotropy in those materials’ ductility [20]. They pointed out that the tensile properties and elongation of the cladding layers can be significantly improved through post-heat treatment. The improvement of tensile properties can be explained by the production of a more favorable microstructure, which led to a more ductile fracture behavior in the region of the cladding layer and the HAZ. With the application of post-heat treatment, the tempered martensite, which was associated with a finer dendritic structure, contributed to the improvement of the ductility, as evidenced by the increased elongations. The ductile behavior was also confirmed in SEM micrographs. Without the post-heat treatment, the fracture surface presented little/no micro-void formation and dimpling, which indicates insignificant plastic deformation and is consistent with a relatively low elongation. On the other hand, dimpling was obvious on the fracture surface of the HAZ of the post-heat specimen, which is consistent with the higher elongation [20].

The existing of the shear stress, which is produced and inevitable during the rail and wheel contact, directly influences the material behavior, i.e., wear or RCF and is one of the vital causes of the rail damage [55]. It could be certified by the appearance of the RCF defects near the locations with high shear stress [56]. Investigation by Lai reported the shear strength of the cladded layers with four cladding materials including 410 L, 420SS, Stellite 6, and Stellite 21 by shear punch tests. The results indicated that the ultimate shear strength (USS) of 410 L cladded rails (791 ± 7 MPa) presented 84% of the substrate (937 ± 8 MPa) and was the lowest value among the four cladding materials owing to the large portion of ferrite in the 410 L microstructure. However, the USS of 420SS showed the highest value (1407 ± 29 MPa) and greater than that of the untreated rails, contributing to the fully martensitic dendrites in the microstructure. In the case of Stellite 6 and Stellite 21, the USS values (909 ± 19 MPa and 1005 ± 20 MPa, respectively) were comparable to that of the substrate that might be caused by the solid-solution strengthening effects of the metal carbides [3].

In fact, the factors that can lead to the failure and fracture of rails are not only the alternating normal and tangential stress but also the bending stress created by the vertical load [57,58]. Therefore, the effects of cladded coating on the bending properties and fracture behavior of rails have attracted the attention of researchers. Li et al. reported that the U71Mn rail cladded by Ni-based alloy powder presented much better bending properties with higher ultimate bending strength (1578 ± 12 MPa) and fracture strain (3.81 ± 0.05%) than that by laser cladding without heat treatment (1172 ± 18 MPa and 0.89 ± 0.01%, respectively). The superior bending properties contributed to the microstructure and performances in the HAZ where the fine pearlite microstructure contributed to promote the bending strength and bending toughness. During the laser-cladding process without heat treatment, the HAZ, in which martensitic structure with low plasticity and low fracture toughness is presented, induces the generation of damages and increases the extending rate of cracks under the bending stress [59].

### 3.2. Fatigue Resistance

**Figure 5:**
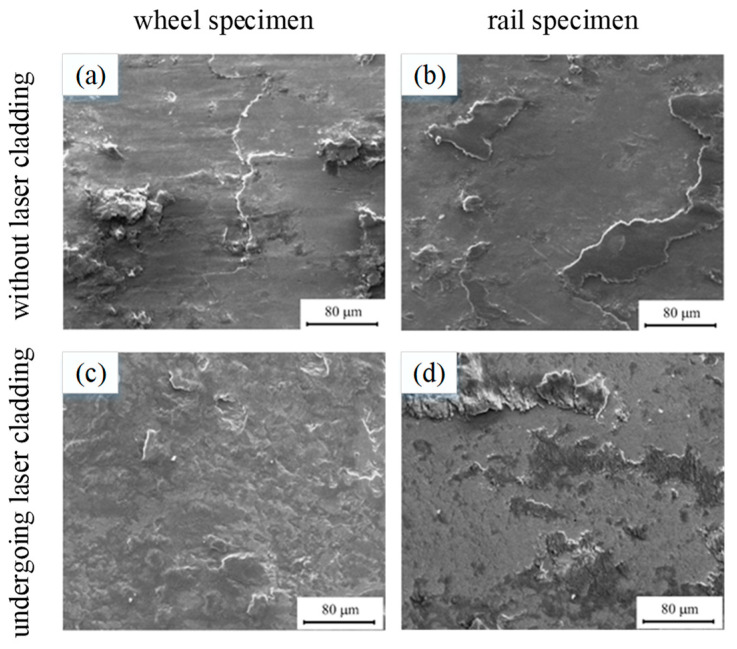
SEM morphologies of (**a**) wheel and (**b**) rail specimens without laser cladding (25 t), (**c**) wheel and (**d**) rail specimens undergoing laser cladding with common Co-based alloy powders (Reprinted from ref. [31], copyright (2014), with permission from Elsevier).

In the situation of a wheel/rail without laser cladding, the wear mechanism is adhesion wear and serious surface spalling, as shown in Figure 5a,b, which were obtained from the heavy-haul wheel/rail materials. The laser cladding coatings would markedly decrease weight loss from wear of wheel/rail. Based on the work reported by researchers [31], the laser cladding coatings would effectively improve the wear resistance of wheel/rail and reduce the wear loss of heavy-haul wheel/rail. Compared by the wheel/rail without laser cladding, the decrease rates of weight loss of wheel and rail rollers are 78.8% and 78.5% [31]. In addition, owing to the high surface hardness of the coating, the wear mechanism is plowing, small spalling, and abrasive wear, which contribute to the slight spalling on the wear surface, as shown in Figure 5c,d which were obtained from the Co-based alloy cladded layer on the heavy-haul wheel/rail materials. Wang pointed out that the hard carbide Cr_23_C_6_ of the Co-based alloy coating has excellent wear resistance and leads to slight surface damage of the wheel/rail [31,60].

For the rail/wheel during service life, the primary problems to be prevented are wear and fatigue crack growth, which can result in the rail breaking if they are not restrained [61,62]. Crack initiation and propagation follows a number of stages [63]. Crack initiation is driven by a ratchetting process. Owing to the contact stresses, the cracks will propagate until the cracks turn down and develop rapidly, which contributes to bending [25,63]. Laser cladding presents a favorable option for improving the fatigue resistance through depositing a high-quality coating with less porosity, more homogeneity, and superior properties on the rail/wheel surface. In addition, the cladded layers have much more tolerance to the high heat input from sliding and tread breaking, which can change the residual stress below the surface to tensile. As we know, the residual stress has a significant effect on the crack growth. The tensile residual stress is proved to be harmful for the wear and fatigue resistance, but the compressive residual stress is a benefit [64]. Despite the complex geometric features of the material, including the substrate, the HAZ, and the clad, the fatigue behavior presents a remarkable low scatter. The fatigue behavior of the cladded material is mainly determined by the properties of the HAZ, whereas the fracture does not stem from this zone.

**Figure 6:**
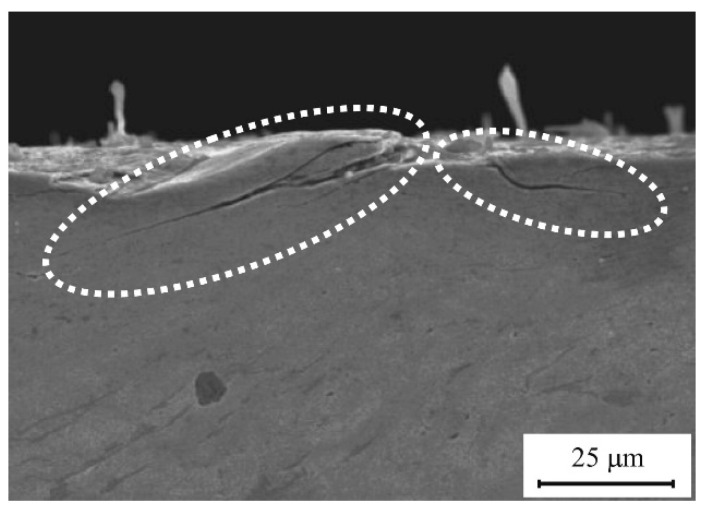
Fatigue crack of wheel/rail specimens without laser cladding (axle load: 25 t, cycle number: 4.8 × 105, Reprinted from ref. [31], copyright (2014), with permission from Elsevier).

The status of surface stress of a single-track clad presents compressive stress that contributes to preventing the initiation of the crack. However, during the laser-cladding process conducting multi-layer or multi-track cladding, the status of surface stress would change from compressive stress to tensile stress. A crack is inclined to occur at the region of the grain boundary, impurity, and pore etc. in which the strength is relatively weak and the concentration of stress would be easily induced. Based on the region of crack initiation, the crack might initiate from the surface of the substrate, inside of the clad, or the region of overlapping that is closely relevant to the crack-resistant areas of the regions dominated by the physicochemical characteristics of the clad and substrate. In addition, the fatigue cracks are easy to initiate in both the surface and subsurface due to large contact stress (Figure 6) [31]. Clare et al. also pointed out that a lower scanning speed and higher powder flow rate, which accompanied the increase of energy density of the laser beam, would lead to the initiation of cracks owing to the coefficient of thermal expansion mismatch between the clad layer and the substrate and internal stress in the coating [45,65,66]. The results reported by Wang indicated that the wheel/rail substrate would have obvious plastic deformation under the heavy-haul condition. However, the laser-cladded layer would exhibit streamline deformation, which testifies that the laser-cladding coating can restrain the crack initiation and alleviate surface damage [31].

**Figure 7:**
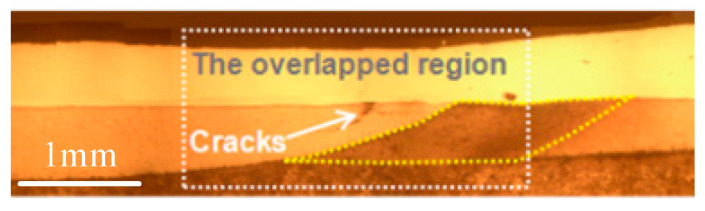
Cross-section macrographs of the two-layer Ni-based alloy cladding tracks on U71Mn rail by laser cladding (Reprinted from ref. [26], copyright (2019), with permission from Elsevier).

When the cladded rail/wheel suffer from the cyclic loading, the different regions, that contain the clad, HAZ, and the substrate would present diverse crack sensitivity. For instance, Niederhauser et al. pointed out that the substrate was the most susceptible to cracking, whereas the cracks stemmed only from the shear bands in the cladded layers and appeared only at the high test strains. In addition, there was only a handful of cracks in the HAZ, and it seems to sustain even the highest test strain amplitudes with the value of 1% [48]. Under circumstance of the two-layer cladding tracks deposited by the Ni-based powder, the cracks originated from the overlapping region of the HAZs and propagated along the direction that is parallel to the interface of the substrate and coating [26], as shown in Figure 7. Lewis et al. carried out laser-cladding tests on the standard R260 grade rail discs, in which six candidate cladding materials including a multi-phase Manganese Steel Variant (MMV), Martensitic Stainless Steel (MSS), TWIP Steel, NiCrBSi, Stellite 12, and Stellite 6 were chosen for the test to assess the wear and RCF performance of the cladded rail. The Stellite 6 deposit with wear rates of less than 70% of a R260 Grade disc was much better than either Stellite 12 or MSS presenting excellent fusion at the HAZ interface and a virtually porosity-free surface [50].

## 4. Process Optimization for Enhanced Mechanical Properties

In the laser-cladding process, the metal material would melt and solidify rapidly. The high cooling rate contributes to the generation of a fine microstructure. However, it would result in high residual stress, which has a significant effect on the mechanical properties. Heat treatment, which is conducted by heating, preservation, and cooling to obtain the prospective microstructures and mechanical properties, was adopted by researchers to modify the characteristics of the cladded rails. Pre-heating, which is the treatment conducted by a laser beam without the participation of powder before the laser-cladding process, could lead to the increase of the dilution ratio of the coating which may have a negative influence on the mechanical and physical properties of the coatings. The fact has been indicated by the results of Li’s investigation, in which the depth of the HAZ and the penetration depth of the Ni-based alloy coating on U71Mn rail significantly increased [26]. Li also investigated the influence of pre-heating and post-heating on the cracking behaviors, microstructures, and mechanical properties of an Ni-based coating on a full-scale rail by laser-induction hybrid cladding in which the pre-heating and post-heating temperatures are 600 °C. The results indicated that the cracking and martensite transformation that occurred in the HAZ can be prevented by post-heating. However, the fine perlite with a smaller perlite block size and lower interlamellar spacing formed [26]. In addition, the investigation by Lai, in which 410 L, 420SS, Stellite 6, and Stellite 21 were selected as cladding material to clad on the rail substrate, reported that the application of pre-heating at 350 °C was insufficient in preventing the formation of martensite in the HAZ of the cladded rails irrespective of the number of cladding layers and cladding materials. However, the combination of pre-heating and post-heating can effectively slow the cooling rate that was proved to provide beneficial tempering to the martensite that formed in the HAZ of the cladded rails [3].

**Figure 8:**
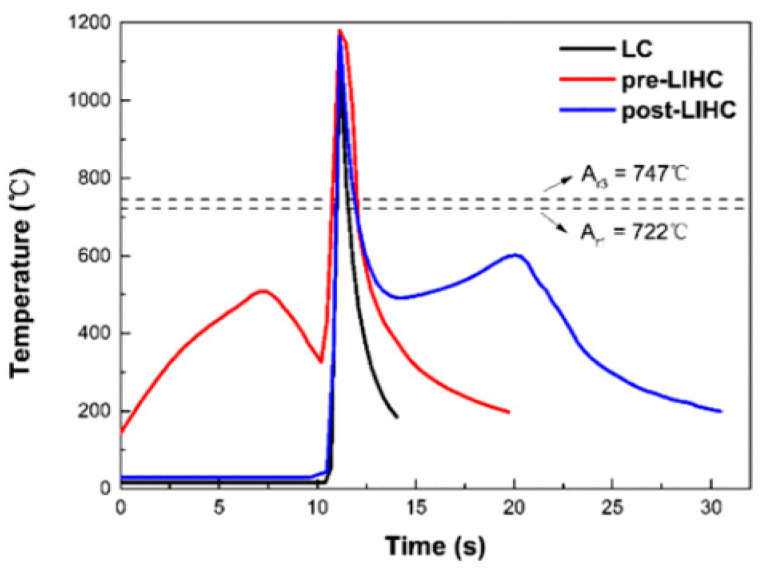
Thermal cycle curves of the heat-affected zones (HAZs) under different technologies (reprinted from ref. [26], copyright (2019), with permission from Elsevier).

The different microstructural characteristics of HAZ under different conditions of heat treatment are related to the relationship between the thermal cycle and microstructure of the HAZ. Based on the solid phase transformation principle of steel materials, the microstructural characteristics of HAZ are mainly determined by the maximum temperature (THAZ) and the cooling rate between 800 and 500 °C [26,67]. However, the fact that THAZ in the HAZ would present above 1100 °C, which is higher than the austenitizing temperature (such as 747 °C of the U71Mn steel), indicated that the austenitized processing has been completed in the heating period. Therefore, the microstructural features of HAZ would be decided by the cooling process. As shown in Figure 8, the THAZ of the HAZs without heat treatment or with pre-heating decreases to 200 °C rapidly. However, the cooling process sustains about 20 s with post-heating treatment; meanwhile, the temperature keeps in the range of 500–600 °C for about 10 s, in which an isothermal transformation process occurs [26]. The 10 s is enough to insure the austenite transform to pearlite drastically in the HAZ by post-heating that can explain why there is no martensite, which would induce high hardness, high brittleness and low toughness [68]. Due to the fine pearlite structure, which is accompanied by high plasticity and fracture toughness in the HAZ by post-heating treatment, the HAZ significantly promotes both the critical stress of crack propagation and bending strength, which would change the fracture mechanism [59].

The other method to enhance the properties of the coating on the rail is mixing the element that has a reinforcing effect into the cladding material. For instance, Wang added the lanthanum oxide (La_2_O_3_) to Fe-based alloy powder with different mass fractions to create the cladded layers on the wheel and rail rollers using a multimode cross flow CO_2_ laser (TR-3000). The results indicated that the lanthanum oxide has a positive influence on the refining of the microstructure due to the activity of the La element, which improved the wear resistance [69].

In addition, some considerable treatments that were used in other applications of laser cladding or other additive manufacturing technology might be suitable methods to enhance the mechanical properties of the wheel/rails by laser cladding. For instance, Duarte et al. conducted the production of samples of 316 L stainless steel and proposed a new hot forging wire and arc additive manufacturing (HF-WAAM), in which the material is locally forged immediately after deposition by WAAM, resulting in the occurrence of the in situ viscoplastic deformation at high temperatures. Afterwards, the microstructure is refined because of the recrystallization of the previous solidification structure in the subsequent layer deposition. The yield strength and UTS are improved as well, as the pores are reduced, which is attributed to the hot forging process [70]. Donoghue et al. combined a rolling step sequentially with layer deposition in the production of Ti–6Al–4V by WAAM to promote the refinement of β grain because the primary β grain structure would result in a strong texture and mechanical anisotropy in AM components. With the application of rolling after each added layer deposition, only a surprisingly low level of rolling deformation is required to reduce the β grain size to < 100 μm [71]. On the other hand, additional physical fields such as electric field and magnetic field have been combined with the laser cladding to enhance the mechanical properties. For instance, Liu et al. cladded the Fe 60 on the Q 235 steel by rotating the magnetic field auxiliary laser cladding. The results indicated that the microstructure is refined, and the hardness of the coating increases about 10% compared with that deposited without magnetic filed [72]. Chen et al. deposited the 304 coating on the #45 steel by the laser cladding with the auxiliary of an electric and magnetic coupled field. The electromagnetic stirring created by the magnetic filed is strengthened with the presence of the electric field, which was also verified in other investigation [73,74]. The coating presents obvious refined microstructure, higher hardness, and improved wear resistance compared with the coating by laser cladding without auxiliary [75].

## 5. Conclusions

In this paper, the research status of microstructural and mechanical characteristics of cladded rails by laser cladding have been reviewed. The main conclusions are drawn as follows:

(1) The effects of processing parameters (e.g., laser power, scanning speed, powder feed rate, etc.) on the cladded layer have been reviewed, indicating that a proper selection of process parameters contributes to controlling the geometry of the cladded layer, obtaining the fine dendritic morphology and reducing the defects (heating bubbles, for instance).
(2) The overview images of all microstructures would show an HAZ dilution zone, coarse-grained zone, and fine-grained microstructure zone, followed by an inter-critical or sub-critical HAZ adjacent to the base material based on the difference of the substrate. In addition, the brittle martensitic structures, which are harmful to the mechanical properties, would be formed in the HAZ.
(3) With an increase of depth, the hardness of the cladding coatings decreases gradually along the cladding layer and finally close to the hardness of substrate. However, the HAZ region would present much higher hardness than that of the substrate and coatings because of the existence of the martensite structure. With the difference of the cladding material, the distribution of residual stress will be different.
(4) The cladding layers would present similar or higher yield strength and UTS but lower elongation values than the non-cladded rail steel regardless of the cladding material. The tensile properties, elongation, and bending properties of the cladding layers can be significantly improved through post-heat treatment owing to the production of a more favorable microstructure, which led to a more ductile fracture behavior in the region of cladding layer and the HAZ.
(5) The clad, HAZ, and the substrate would present diverse crack sensitivity. The fatigue behavior of the cladded material is mainly determined by the properties of the HAZ, whereas the fracture does not stem from this zone.
(6) In order to restrain the martensitic transformation and ultimately enhance the properties of the cladded layer, conducting heat treatment and mixing strengthening element into the cladding material are popular methods to optimize the laser-cladding process.

## Figures and Tables

**Table 1:** Summary of materials used in cladded of rails by laser cladding.

Substrate	Powder		Process Parameters			Ref.
			Laser Powder (W)	Scanning Speed (mm/s)	Powder Feed Rate (g/min)	
Hyper-eutectoid rail CL60 (GB) wheel U71Mn (GB) rail R400HT (EN) HE400 (EN) R260 (EN) R200 (EN)	Fe-based	316 L, 410 L 410, 420, 421	3200	16.7–20	3RPM	[3,17,18,19,20,21]
			500	10/8	1.2	
		MSS				[7,22,23,24]
		TWIP steel				[23]
	Ni-based	NiCrBSi, Inconel 625, Hastelloy C, Nickel alloy	1200–1800	6.67–15	15–30	[22,25,26,27,28]
			4500	16.7	18	
	Co-based	Stellite 6 Stellite 21	1600	6.67–13.33	15–30	[3,17,22,23,29,30,31]
			1200–1800	6.67–15	15–30	
	Other	Ti, TiB_2_	1500	16.7	2	[32]

**Table 2:** Average tensile properties with a standard deviation of the longitudinal specimens from cladded hypereutectoid steel rails (HE400) with different cladding materials [20].

Cladding Material	Sampling Position	Yield Strength (MPa)	UTS (MPa)	Elongation (%)
401 L	Cladding layer	910 ± 4.1	1149 ± 12.5	3 ± 0.2
	HAZ	1060 ± 8.1	1317 ± 6.2	4.32 ± 0.02
	Substrate	1000 ± 8.1	1299 ± 4.7	5.8 ± 0.04
SS420	Cladding layer	800 ± 10.3	1455 ± 19.4	1.4 ± 0.3
	HAZ	910 ± 5	1156 ± 3	10.2 ± 0.05
	Substrate	880 ± 0	1240 ± 10	8.8 ± 0.1
Stellite 6	Cladding layer	925 ± 4.7	1302 ± 47.6	2.2 ± 0.2
	HAZ	855 ± 15	1092 ± 4	10.2 ± 0.1
	Substrate	910 ± 12.5	1262 ± 2.5	7.8 ± 0
Non-cladded rail steel		917 ± 13.88	1288 ± 6.24	8.27 ± 0.22

## Data Availability

Data is contained within the article.
